# Trait Forgiveness Moderated the Relationship Between Work Stress and Psychological Distress Among Final-Year Nursing Students: A Pilot Study

**DOI:** 10.3389/fpsyg.2020.01674

**Published:** 2020-07-09

**Authors:** Lingyan Li, Caixia Yao, Yan Zhang, Guangyuan Chen

**Affiliations:** ^1^School of Nursing, Nanchang University, Nanchang, China; ^2^Department of Nursing, Jiangxi Health Vocational College, Nanchang, China; ^3^The Second Clinical Medical College, Nanchang University, Nanchang, China

**Keywords:** trait forgiveness, nursing work stress, psychological distress, nursing student, moderator

## Abstract

This study was to explore the potential moderating effect of trait forgiveness and its facets on the relationship between perceived work stress and psychological distress among Chinese nursing students in clinical practice. A total of 182 Chinese nursing students who had been receiving final-year clinical training completed self-report measures of nursing work stress, trait forgiveness and psychological distress. Correlation analysis and hierarchical multiple regressions were mainly applied for data analysis. Results showed that trait forgiveness was negatively associated with psychological distress, even after controlling for the effects of perceived work stress and demographic/workplace related variables. Further analyses indicated that the ability to forgiveness of situations was particularly crucial in reducing the negative effects of perceived work stress on psychological well-being, especially when students perceived higher level of stress. These results demonstrated that alternative interventions targeting on trait forgiveness, especially those programs which can improve one’s ability to acceptance uncontrollable bad circumstances, may be beneficial for the well-being of nursing students in clinical practice.

## Introduction

Numerous studies have revealed that nursing students present various degree of stress. A high level of stress may result in impaired psychological well-being such as depression, anxiety, burnout syndrome, and in extreme cases, post-traumatic stress disorder among these students, especially in the stage of clinical practice (Smith and Yang, 2017; Admi et al., 2018). The stressor of clinical placement may be stem from several facets. For example, working with dying patients, insecurity of clinical competence, fear of making mistakes and interpersonal conflicts with patients and staff have been reported by student nurses as stressors associated with the clinical environment (Jackson et al., 2011). Specifically, lack of knowledge and skills and fear of medical error resulted from inadequate preparation might be the two main stressors. It was found that perceived stress in the 4th year nursing students was at pretty much the same level of that in newly graduated nurses, but nursing students scored high in inadequate preparation factor while new nurses scored high in the workload factor (Suresh et al., 2013). A recent study reviewed the previous research and showed that nursing students perceived moderate to high levels of stress during their clinical trainings. Among these stressors, lack of knowledge and skills is reported as a frequent stressor, sometimes is the main clinical stress (Bhurtun et al., 2019). Similarly in China, newly graduated nurses working in a pharmacy admixture service reported that lack of professional nursing competence was the main source of their stress followed by heavy workloads and the sociological environment (Wang et al., 2015). Although the relationships need further exploration (Labrague and McEnroe-Petitte, 2018), evidence show that some individual and organizational factors such as female gender, low family income, and shift might contribute to nurses’ stress experiences and well-being (Jones et al., 2015; Alzayyat and Al-Gamal, 2016).

It may be unrealistic to remove each stressors related to clinical practice for every nursing student, whereas it is indeed feasible to reduce the strong influence of nursing work stress. According to Folkman et al.’s stress and coping theory (Folkman, 1984), whether and to what extent perceived stress is predictive of negative psychological outcomes depends on not only the characteristics of the stressors, but also the individual’s appraisal of these stressors and their access to coping resources. Hence, identifying coping resources that may hinder stress management or better enable students to cope with their work environment and reduce stress is the place of key. Exiting evidence have showed that when negative events happened in clinical practice (e.g., medical errors or did clinical task not achieving the expectation, or patients died), students often use maladaptive coping strategies such as self-blame, blame of impersonal forces or the “system,” and blame of others (Collins et al., 2009), which in turn add their psychological stress, even lead to mental illness (Al-Dubai et al., 2011; McMeekin et al., 2017; Ludwig et al., 2019).

Continuous advances have arisen in developing measures of stress management, and new measures based on positive psychology received special attention as a modern way to relieve stress. Previous studies have postulated that positive inner factor such as self-efficacy, resilience and mindfulness would have a positive effect on psychological well-being (He et al., 2018), and these positive psychological traits may moderate the negative effects of appraised stress (Li et al., 2017; Smith and Yang, 2017; Yao et al., 2018; Lu et al., 2019). Thus, researchers have done a lot of work on mindfulness based interventions for nurses, with studies in this field consistently reporting that mindfulness significantly decrease nurses’ work stress and improved nurses’ mental health (Guillaumie et al., 2017). However, the effectiveness of Mindfulness-based interventions need backed up by trained instructors, who are not always accessible in the real world. Watanabe et al. (2019) tried to carry out a brief Mindfulness-based intervention conducted by trained senior nurses, the outcomes showed that the additive value of the brief mindfulness-based stress management program was not confirmed in mental state and self-evaluated work efficiency among nurses.

In particular, a systematic review of interventions to reduce stress in nursing students concluded that stress interventions should be theory based and multi-faceted, focus on interface, including skills for dealing with stressful situations and changing problematic thoughts (Galbraith and Brown, 2011). Forgiveness, which is one of the characteristics of positive psychology that closely related to both blame and mindfulness, is another potential protective factor for stress (Oman et al., 2008). Although dispositional forgiveness has been a research subject of well-being among students in recent years (Hirsch et al., 2011; Khodabakhsh and Mansuri, 2012), there is scarcity of evidence examining the role of forgiveness on work stress among nursing students in clinical practice. What’s more, given that the complexity of workplace in clinical nursing, the two facets that self-forgiveness and forgiveness of others focused in previous studies may not cover all the psychological progress related to forgiveness of negative events in nursing students’ practice.

Therefore, the current study aimed to explore the potential effect of self-forgiveness, forgiveness of others, and forgiveness of situation on working stress in the context of clinical training among Chinese nursing students. Traditionally, nursing clinical placements do not take place in China until the final year. According to the criteria in China’s medical education for undergraduates, nursing students should complete a clinical practice no less than 40 weeks before graduation. Transitioning from the role of nursing student to professional nurse is a complex and multifaceted experience. This time has been identified as a potentially a stressful time for students (Chen and Hung, 2014). This present study pursues two specific goals: first, to test how work stress, trait forgiveness, and psychological distress are interrelated in final-year Chinese nursing students, second, to detect whether trait forgiveness moderates the relationship between perceived work stress and the related psychological health outcomes mentioned previously. Moreover, out of the studies on forgiveness, there have been few studies examining the stress-moderating role of all three forgiveness facets illustrated by Thompson et al. (2005). Therefore, this study will explore the moderating effects of self-forgiveness and forgiveness of others, and forgiveness of situation on perceived work stress and psychological distress. Based on the evidence as aforementioned, we hypothesize that (1) High levels of trait forgiveness would be associated with better psychological well-being for nursing students, and trait forgiveness would moderate the relationship between perceived work stress and psychological well-being. (2) Compared to nursing students with lower levels of forgiveness, the association between higher perceived work stress and more psychological distress would be weaker among the nursing students with higher levels of forgiveness.

## Materials and Methods

### Participants

From February 2019 to April 2019, final-year nursing students in clinical practice were recruited from two general hospitals in Nanchang, Jiangxi Province. Eligible students should meet the following criterion: (1) aged 18 and above; (2) have been practicing in clinical for more than 5 months (half of the minimum time needed); (3) never been diagnosed with depression, anxiety and other mental illness that would interfere with completion of the measures.

### Sample Size

G^∗^Power 3 software (Faul et al., 2007) was used to estimate the sample size needed for multiple regression analysis. With a significance level of 0.05, a medium effect size of 0.15, power of 95.0%, and 11 predictors (i.e., four demographic and four workplace characteristics, work stress, trait forgiveness, and work stress × trait forgiveness), a minimum sample of 178 was calculated. In consideration of 10% possible dropouts, the sample size was increased to 198.

### Measures

#### Demographic and Workplace Information

Participants provided demographic information including age, gender, objective socioeconomic status (marital and education status of parents, and number of siblings). Using the 2-item Subjective Socioeconomic Status Scale, subjective socioeconomic status of participants were also been rated (Hu et al., 2012). In terms of workplace information, number of internship months completed, shift work during practice, and work load were reported. In addition, conflict events experienced or witnessed by participants were collected by 4 questions such as “Have you ever experienced conflict situation with patients during your clinical practice?” Participant may answer none, sometimes, often, and almost all the time according to the occurrence frequency. If participants give a positive answer, then they need to clarify which type situation they have experienced (e.g., oral conflict or physical altercations or sexual harassment or other situation).

#### Work Stress

Work stress was assessed by the 34-item Nursing Stress Scale (NSS) (Gray-Toft and Anderson, 1981). The scale contains 7 subscales assessing different facets of stressful situations experienced by hospital nurses. That is, 7 items on the stressful events related to death and dying patients (e.g., watching a patient suffer), 6 items for workload (e.g., not enough staff to adequately cover the unit), 5 items on scale for conflict with physicians (e.g., disagreement concerning the treatment of a patient), 5 items for conflict with other nurses (e.g., conflict with a supervisor), 5 items for uncertainty concerning treatment (e.g., uncertainty regarding the operation and functioning of specialized equipment), 3 items for inadequate preparation (e.g., being asked a question by a patient for which I do not have a satisfactory answer), and 3 items for lack of staff support (e.g., lack of an opportunity to talk openly with other unit personnel about problems on the unit). Participants rate on a four-point Likert scale (1 = never to 4 = very frequently), with a higher score indicating a higher frequency of experiencing nursing work stress. The Chinese version of NSS showed good internal reliabilities (Lee et al., 2007).

#### Trait Forgiveness

Trait forgiveness was measured by the 18-item Heartland Forgiveness Scale (HFS) (Thompson et al., 2005). HFS is the first tool to measure dispositional forgiveness of self, others, and situations simultaneously. Each subscale contains 6 items, three of which are reverse-coded. Participants rate their agreement with each statement on a 7-point scale ranging from 1 (almost always false to me) to 7 (almost always true to me). Example items for these three subscales are as following respectively: “Although I feel bad at first when I mess up, over time I can give myself some slack” (self-forgiveness); “With time I am understanding of others for the mistakes they’ve made” (interpersonal forgiveness); “Eventually I let go of negative thoughts about bad circumstances that are beyond anyone’s control” (forgiveness of situation). Scores are calculated by summing the items on each scale, with the negatively worded items being reverse scored. A higher score represents more prone to forgive. The Chinese version HFS has been applied widely and showed good reliability and validity (Zhang, 2009; Chan, 2013).

#### Psychological Distress

Psychological distress was evaluated using the 21-item Version of the Depression Anxiety Stress Scale (DASS-21) (Antony et al., 1998). The DASS-21 contains three subscales evaluating and distinguishing three areas of distress: depression symptoms (e.g., I felt that life was meaningless), anxiety symptoms (e.g., I felt scared without any good reason), and general stress (e.g., I found it hard to wind down). Each subscale comprises 7 items, and participants rate the applicability of each item to the past week on a scale from 0 (Not at all) to 3 (Most of the time). Higher scores mean more severe symptoms of psychological distress. The DASS-21 has been widely used across cultures (Scholten et al., 2017). The Chinese version of DASS-21 showed good psychometric evidence (Wen et al., 2012).

### Data Collection

Eligible nursing students were invited to participate in this survey through the WeChat (an instant messenger App) group, which was set to facilitate the communication among students receiving nursing clinical training in the same hospital. The digital questionnaires were distributed as a web page link to the online questionnaire survey site^1^ through the WeChat to students’ smart phone. When a potential participant received an invitation on their phone, they were able to choose to participate or to reject, after been informed of the study purpose and participating criterion by a separate webpage. Once a nurse student chose to participate, she/he provided a digital informed consent by clicking a box in the webpage, and also claimed that they meet the criterion. Participants completed and submitted the questionnaires in 2 weeks since the day they were issued. The website for the online survey recorded their information. To address any perception of a power differential between the researchers and participants, no identifying data were collected. We set an anti ‘survey replication’ option of the website which records cookies and restricts multiple attempts at the survey.

### Data Analysis

SPSS 18.0 were used to do data management and most of the data analysis. The demographic status and workplace condition, work stress, psychological distress, and trait forgiveness were reported through descriptive statistics. By using correlational analysis, the correlations among work stress, psychological distress, and trait forgiveness were assessed. Hierarchical multiple regression analyses were conducted to test potential interactive effect of trait forgiveness with work stress, as well as the three facets individually, in the prediction of psychological distress. The independent variables were entered in the following steps: (a) the controlled demographic and workplace related variables, (b) the total perceived work stress by nursing students, (c) the total trait forgiveness, and (d) the interaction term of work stress × trait forgiveness. Trait forgiveness was entered after work stress to explore the unique differences in psychological distress in the test results. Centered scores were used for the interaction terms to avoid multicollinearity. To explore the potential main and moderate effects of the three forgiveness facets, regression analyses were conducted repeatedly using the three forgiveness facets to replace total forgiveness at step 3, and three interactions between each facet and the work stress were input in step 4a.

As the test of skewness (0.198–1.363) and kurtosis (0.339–1.975) indicated non-normality of some variables in our data and the relative small sample size of this study, more robust analysis of the direct and indirect effects was needed. Bootstrap confidence intervals (CI) of moderation analysis model results were obtained by using the BOOTSTRAP option in Mplus 8.2 (Muthén and Muthén, 1998-2017).

Simple slope analyses were conducted to illustrate the significant interactions among all the moderating models. According to Jaccard et al. (2003), the unstandardized regression coefficients (B) of the regression lines for nursing students for low (1 SD below mean) and high (1 SD above mean) effects of the moderating variable were adopted. A *p*-value of 0.05 was considered to have statistical significance.

## Results

### Description Statistics

#### Demographic and Workplace Information About the Participants

Of all the 198 nursing students invited to this study, 182 agreed to participate and successfully completed the survey. The age of 182 students ranged from 19 to 24, with a mean age (*SD*) = 21.66 (0.84) years. Majority of these students were female (89.0) and had more than 1 sibling (86.8). Averagely, the nursing students had finished 9.71 months of clinical training, and 74.2% of them need to shift, with an average of 30% proportion to the whole duration. What’s more, about two thirds and a half of students had experienced and witnessed conflicts with patients respectively. The more details of demographic and workplace information about the participants was present in Table 1.

**TABLE 1:** Socio-demographic and workplace characteristics of participants (*N* = 182).

	*M* ± *SD*	n	%
Age (years)	21.66 ± 0.84		
Subjective socioeconomic status	11.68 ± 2.25		
**Gender**			
Male		20	11.0
Female		162	89.0
**Number of siblings**			
None		24	13.2
≥1		158	86.8
**Marriage status of parent**			
Married		174	95.6
Divorced		4	2.2
Widowed		4	2.2
**Education level of father**			
Primary school		43	22.6
Junior high school		82	45.1
Senior high school		37	20.3
College and above		20	11.0
**Education level of mother**			
Primary school		76	41.8
Junior high school		76	41.8
Senior high school		19	10.4
College and above		11	6.0
Duration of clinical practice (months)	9.71 ± 0.63		
**Whether on shifts**			
Yes		135	74.2
No		47	25.8
Proportion of shifts on duration	30.08 ± 14.05		
Work hours per week	40.84 ± 3.27		
Number of patients in charge per day	11.90 ± 3.99		
**Experienced conflicts with patients**			
Sometimes		122	67.0
Never		60	33.0
**Witnessed conflicts with patients**			
Sometimes		82	45.1
Never		100	54.9

As can be seen from Table 2, Inadequate preparation (Mean of average item score = 2.18) and Uncertainty concerning treatment (Mean of average item score = 2.08) were the top two stressors perceived by nursing students, followed by Death and dying (Mean of average item score = 1.96). Conflict with physicians emerged as the least stressor, followed by Workload, with a mean of average item score was 1.64 and 1.75, respectively. Mean scores of the three facets associated with dispositional forgiveness were from 26.30 (self forgiveness) to 28.62 (forgiveness of others). Participants’ scores on psychological distress were averagely 3.27 (depression symptoms), 4.24 (anxiety symptoms) and 4.56 (general stress), from low to high. According to the cutoff of DASS-21 (Brown et al., 1997), results also indicated that 5.5% (10/182) 0f these students were positive for depression symptoms, 25.8% (47/182) were positive for anxiety symptoms, and 12.1% (22/182) had positive symptoms of general stress.

**TABLE 2:** Participants’ respondents on NSS, HFS and DASS-21 (*N* = 182).

	Range	*M* (*SD*) of factor score	*M* (*SD*) of average item score
**NSS**			
Death and dying	6–20	11.78 ± 2.34	1.96 ± 0.39
Conflict with physicians	6–19	9.85 ± 2.45	1.64 ± 0.41
Inadequate preparation	4–14	8.74 ± 1.77	2.18 ± 0.44
Conflict with other nurses	3–10	10.48 ± 2.55	1.82 ± 0.53
Workload	6–21	10.41 ± 2.76	1.75 ± 0.42
Uncertainty concerning treatment	5–31	7.36 ± 1.93	2.08 ± 0.55
Lack of support	4–14	5.47 ± 1.58	1.84 ± 0.48
**HFS**			
Self forgiveness	18–39	26.30 ± 3.29	4.38 ± 0.55
Forgiveness of others	21–41	28.62 ± 3.49	4.77 ± 0.58
Forgiveness of situations	17–41	27.45 ± 3.98	4.58 ± 0.66
**DASS-21**			
Depression symptoms	0–17	3.27 ± 3.54	0.47 ± 0.51
Anxiety symptoms	0–18	4.24 ± 3.33	0.61 ± 0.48
General stress	0–17	4.56 ± 3.74	0.65 ± 0.53

### Correlations Among the Study Variables

Table 3 shows the correlations between the study variables. Each subscale of psychological distress had a significant moderate positive correlation with work stress (*r* = 0.433–0.490, *p* < 0.01), and was significantly negative correlated with the overall trait forgiveness at a moderate level, with *r*-values ranging from −0.334 to −0.405. Besides, results confirmed a significant negative correlation between work stress and trait forgiveness (*r* = −0.353, *p* < 0.01).

**TABLE 3:** Correlations among work stress, trait forgiveness, and psychological distress.

	1	2	3	4	5	6	7	8
1. Self forgiveness	–							
2. Forgiveness of others	0.122	–						
3. Forgiveness of situations	0.409**	0.421**	–					
4. HFS_total	0.667**	0.696**	0.849**	–				
5. NSS_tatal	−0.272**	−0.217**	−0.294**	−0.353**	–			
6. Depression symptoms	−0.321**	–0.037	−0.375**	−0.334**	0.433**	–		
7. Anxiety symptoms	−0.333**	–0.082	−0.397**	−0.370**	0.443**	0.850**	–	
8. General stress	−0.384**	–0.119	−0.392**	−0.405**	0.490**	0.828**	0.830**	–
9. DASS-C21_total	−0.368**	–0.085	−0.411**	−0.393**	0.483**	0.946**	0.943**	0.942**

**p < 0.01. HFS_total is total score on Heartland Forgiveness Scale, NSS_total is total score on Nursing Stress Scale, and DASS-C21_total is total score on the 21-item Version of the Depression Anxiety Stress Scales.

In terms of the relationship between the demographic/workplace characteristics and the study variables, results showed that higher level of work stress was associated with worse subjective socioeconomic status (*r* = −0.156, *p* < 0.05), more work hours per week (*r* = 0.211, *p* < 0.01) and conflict events experience (67.95 vs. 62.19, *p* = 0.002). Higher level of trait forgiveness was only associated with better subjective socioeconomic status (*r* = 0.214, *p* < 0.05), whereas more psychological distress was only associated with more work hours per week (*r* = 0.206, *p* < 0.01).

### Moderating Effects of Trait Forgiveness on Psychological Distress

Table 4 demonstrates the four models used to test the main effect and joint effects of work stress and trait forgiveness on psychological distress. In model 1, the inclusion of three related demographic/workplace variables resulted in work hours per week as a positive predictor of psychological distress (*B* = 0.630, *p* = 0.006). In model 2 and model 3, after controlling for the demographic/workplace variables, the entry of work stress and the total trait forgiveness indicated that work stress was positively associated with psychological distress (*B* = 0.434, *p* < 0.001), whereas trait forgiveness showed a protect role on psychological health (*B* = −0.343, *p* < 0.001). The work stress and the total trait forgiveness could account for 23.5 and 6.4% of the variance of psychological distress, respectively. In model 4, after the entry of the interaction term (work Stress × total trait forgiveness), both the main effect and joint effect of work stress and the total trait forgiveness turned out to be not significant. In step 3a, when the total trait forgiveness was replaced by the three facets of forgiveness, results showed negative effect of self forgiveness (*B* = −0.482, *p* = 0.017) and forgiveness of situations on psychological distress (*B* = −0.754, *p* < 0.001), but not forgiveness of others (*B* = 0.343, *p* = 0.072). In step 4a, after the entry of the three interactions between work stress and trait forgiveness facets, the main effects of work stress and forgiveness facets were also not significant, but one moderating effect was found. Forgiveness of situations moderated the relationship between work stress and psychological distress among nursing students (*B* = −11.245, *p* = 0.030). The Bootstrap analysis conducted by Mplus showed that these above main effects and interact effects were stable. The 90% CIs of main effects were (*B* = 0.205–0.426, *p* < 0.001), (*B* = −0.448 to −0.167, *p* < 0.001), (*B* = −0.788 to −0.163, *p* = 0.021), and (*B* = −1.167 to −0.436, *p* < 0.001) for work stress, total trait forgiveness, self forgiveness and forgiveness of situations respectively. The 90% CI of interact effect for forgiveness of situations ^∗^ work stress was (*B* = −0.058 to −0.001, *p* = 0.042). Both the main effect of forgiveness of others and the rest of interact effects were not significant.

**TABLE 4:** Hierarchical multiple regression analyses for testing moderation effects of trait forgiveness on work stress and psychological distress.

	Model 1	Model 2	Model 3	Model 3a	Model 4	Model 4a
						
	B, *p* =	B, *p* =	B, *p* =	B, *p* =	B, *p* =	B, *p* =
**Step 1**						
SES	0.308, *p* = 0.348	0.665, *p* = 0.022	0.825, *p* = 0.004	0.810, *p* = 0.003	0.770, *p* = 0.008	0.775, *p* = 0.005
Work hours per week	0.630, *p* = 0.006	0.326, *p* = 0.108	0.270, *p* = 0.165	0.278, *p* = 0.139	0.270, *p* = 0.165	0.261, *p* = 0.161
Conflict events experience	−0.657, *p* = 0.677	−2.742, *p* = 0.051	−2.685, *p* = 0.046	−1.915, *p* = 0.143	−2.734, *p* = 0.043	−1.877, *p* = 0.148
**Step 2**						
Work stress		0.434, *p* < 0.001	0.359, *p* < 0.001	0.344, *p* < 0.001	0.753, *p* = 0.081	0.642, *p* = 0.177
**Step 3**						
Trait forgiveness			−0.343, *p* < 0.001		−0.023, *p* = 0.949	
**Step 3a**						
Self forgiveness				−0.482, *p* = 0.017		−0.559, *p* = 0.654
Forgiveness of others				0.343, *p* = 0.072		−0.865, *p* = 0.342
Forgiveness of situations				−0.754, *p* < 0.001		1.280, *p* = 0.177
**Step 4**						
Work stress × forgiveness					−4.503, *p* = 0.356	
**Step 4a**						
Work stress × Self forgiveness						0.541, *p* = 931
Work stress × forgiveness of others						6.905, *p* = 0.165
Work stress × forgiveness of situations						−11.245, *p* = 0.030
*R*^2^	0.048	0.283	0.348	0.403	0.351	0.424
Δ *R*^2^	0.048, *p* = 0.031	0.235, *p* < 0.001	0.064, *p* < 0.001	0.120, *p* < 0.001	0.003, *p* = 0.356	0.021, *p* = 0.101

*SES is subjective socioeconomic status.*

**FIGURE 1:**
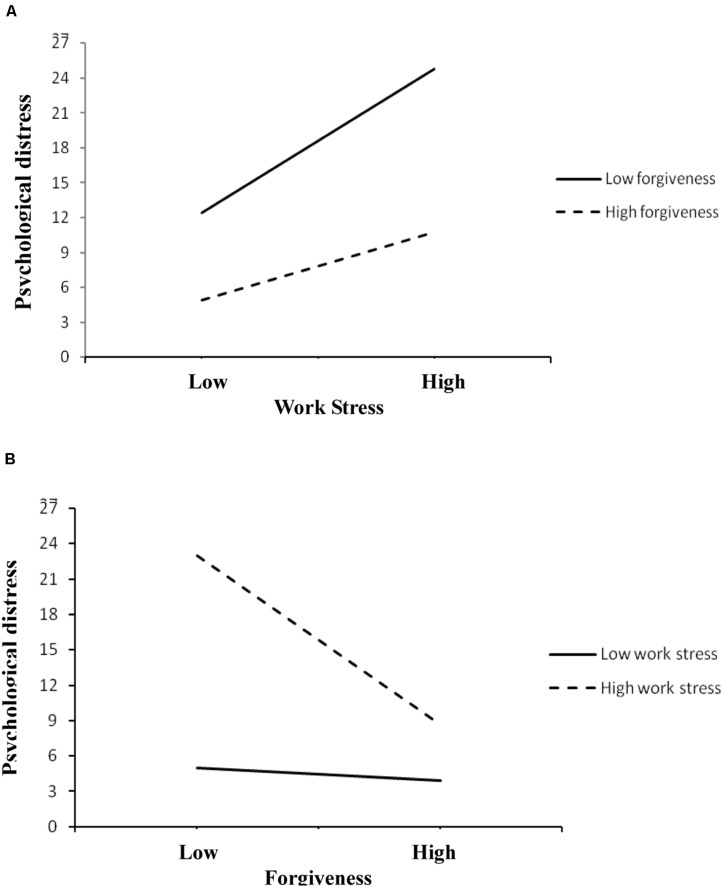
The moderating effect of forgiveness of situations and work stress on psychological distress. **(A)** Displaying association between work stress and psychological distress among students with different levels of forgiveness of situations. **(B)** Displaying association between forgiveness of situations and psychological distress among students with different levels of work stress.

A simple slopes analysis showed that work stress predicted psychological distress at both low (*B* = 0.521, *t* = 3.005, *p* = 0.009) and high levels of forgiveness of situations (*B* = 0.245, *t* = 2.387, *p* = 0.025), but the association between work stress and psychological distress was weaker when the level of forgiveness of situations was higher (see Figure 1A). Similarly, we examined the effect of forgiveness of situations among nursing students with different levels of work stress. As shown in Figure 1B, results revealed that forgiveness of situations buffered the negative effect of stress on psychological health among students with higher level of work stress (*B* = −1.798, *t* = −3.413, *p* = 0.002), but not in the students whose work stress was low (*B* = −0.131, *t* = −0.732, *p* = 0.472).

## Discussion

This study aimed to explore the potential moderating effect of trait forgiveness on the relationships between perceived work stress and psychological well-being in final-year Chinese nursing students. Results of our study showed that the main source of stress related to the clinical practice were Inadequate preparation and Uncertainty concerning treatment, which is consistent with the previous findings that the main clinical stress perceived by nursing students was associated with confidence when caring for patients (McCarthy et al., 2018). This finding implies a need of more simulation training before clinical practice to improve the familiarity with medical language and clinical procedures among the nursing students.

This study found that the overall psychological well-being among nursing students was similar to that in the Chinese normal college students (Gong et al., 2010), and was better than in the nursing stuff (Nie et al., 2013). Unlike findings in previous studies showing the concurrent lower or higher prevalence of psychological problems such as anxiety, depressive symptoms and general stress in nursing students (Chernomas and Shapiro, 2013; Cheung et al., 2016), result in our study revealed that anxiety symptom was the most common problem in these students during their clinical training stage, with over one in four nursing students presenting varying degrees of anxiety, and less students suffering from depression symptoms or general stress. This inconsistency might be due to the special stage in which our data was collected, when the students was facing the challenge both from current clinical studying and graduation thesis soon after. These findings suggest that, during the latter half of the final year, more attention should be paid on the anxiety problems in nursing students.

As hypothesized firstly, the current study confirmed that a higher level of trait forgiveness appeared to be predictive of better psychological well-being, even after controlling for the effects of perceived work stress and demographic/workplace related variables of subjective socioeconomic status, work hours per week and conflict events experience. These findings are in line with existing research showing that the ability of forgiveness may play a buffer role on negative stress responses (Liu et al., 2017) and may serve as a promotion factor for psychological well-being (Chan, 2013). Although the joint effect of work Stress and the total trait forgiveness was not found in this study, results did confirm a joint effect of work Stress and the particular facet of forgiveness (i.e., forgiveness of situations). The association between work stress and psychological distress was weaker in nursing students with higher level of forgiveness of situations than in those at lower level, especially when the level of work stress was perceived as high. This finding supports the second hypothesis of this study, and is in accordance with Thompson et al.’s (2005) finding that, forgiveness of situations was more strongly predictive of psychological well-being above and beyond prediction by self and other forgiveness. Thompson et al. (2005) have proposed that, forgiveness is a coping process enabling individual to resolve the distress induced by events that violate their assumptions, by turning their attention away from these adverse life experiences. Previous study has found that repetitive negative thinking focused on the clinical practice longitudinally predicted the increase of emotional symptoms among novice clinical trainee (Ruiz et al., 2019). To some extent, forgiveness is a form of willing to accept the negative events; it is a sign as psychological flexibility. The difficulty of forgiveness always exhibits as attempt to alter or escape difficult private events in a way called experiential avoidance, a sign as psychological inflexibility (Bond et al., 2011). Thus, to maintain psychological well-being, it is important for nursing students to turn their attention away from those negative situations in clinical practice to more positive aspect of medical workplace. Because that, as we all know, there are still some situations beyond the modern medical technology, even some of which are attributable to medical systems. However, nursing students in this study have reported witness people dying and suffering that beyond their abilities to help as main stressors. Based on findings above, viable interventions designed to improve the ability to forgiveness may be beneficial to reduce or eliminate the negative effects of work stress on the psychological well-being of clinical nursing students. For instance, the acceptance and commitment therapy might be a potential approach (Dereix-Calonge et al., 2019). Also, the workshop plan to improve students’ skills, knowledge, and attitude toward palliative care for dying patients should be considered to be concluded in the training program, which has been evidenced to be useful for the reduction in students’ anxiety and fear of death (Cheong et al., 2020).

To our best knowledge, the present study was the first to explore the relationships among perceived work stress, trait forgiveness, and psychological distress. Our results verified that forgiveness of situation might serve as a protective factor for reducing work stress responses and improving psychological well-being. Besides these strengths, there are also several limitations of this study. Firstly, due to the nature of cross-sectional study design, it is unable to figure out the development of work stress and psychological distress with the progress of clinical practice, it is also unable to draw conclusions about the cause and effect relationships between variables, which need to be further explored through longitudinal studies. Secondly, as a pilot study, we adopted a convenient online survey in a relative small sample, which may contribute to some bias of the study results. A larger scale survey in various hospitals needs to be conducted to extend and generalize the findings in this current study. Thirdly, the trait forgiveness was only measured through questionnaire, future study adopting comprehensive measure approach such as constructive interviews and related experiments will be needed to improve the accuracy of results.

## Conclusion

The relative high prevalence of anxiety symptoms associated with the work stress due to lack of enough knowledge and skills about clinical nursing among final-year students deserves immediate attention. Trait forgiveness has the potential to act as a psychological resource to help individual cope with work stress more adaptively, especially when they perceived higher level of stress. Findings in this study suggest that alternative interventions targeting on trait forgiveness, especially those programs which can improve one’s ability to acceptance uncontrollable bad circumstances, may be beneficial for the well-being of nursing students in clinical practice.

## Data Availability Statement

The datasets generated for this study are available on request to the corresponding author.

## Ethics Statement

The studies involving human participants were reviewed and approved by the medical research Ethics Committee of the first affiliated hospital, Nanchang University. The patients/participants provided their written informed consent to participate in this study.

## Author Contributions

LL conceived and designed the study, analyzed the data, and drafted the manuscript. CY and YZ organized and supervised data collection. CY and GC provided critical comments on various drafts of the manuscript. All authors read and approved the final manuscript.

## Conflict of Interest

The authors declare that the research was conducted in the absence of any commercial or financial relationships that could be construed as a potential conflict of interest.

## Footnotes

1 www.wjx.cn
